# Safety and Efficacy of Denosumab in Children With Osteogenesis Imperfecta—the First Prospective Comparative Study

**DOI:** 10.1210/clinem/dgad732

**Published:** 2024-01-10

**Authors:** Jiayi Liu, Xiaoyun Lin, Lei Sun, Qian Zhang, Yan Jiang, Ou Wang, Xiaoping Xing, Weibo Xia, Mei Li

**Affiliations:** Department of Endocrinology, Key Laboratory of Endocrinology, National Health and Family Planning Commission, Peking Union Medical College Hospital, Chinese Academy of Medical Sciences and Peking Union Medical College, Beijing 100730, China; Department of Endocrinology, Key Laboratory of Endocrinology, National Health and Family Planning Commission, Peking Union Medical College Hospital, Chinese Academy of Medical Sciences and Peking Union Medical College, Beijing 100730, China; Department of Endocrinology, Key Laboratory of Endocrinology, National Health and Family Planning Commission, Peking Union Medical College Hospital, Chinese Academy of Medical Sciences and Peking Union Medical College, Beijing 100730, China; Department of Endocrinology, Key Laboratory of Endocrinology, National Health and Family Planning Commission, Peking Union Medical College Hospital, Chinese Academy of Medical Sciences and Peking Union Medical College, Beijing 100730, China; Department of Endocrinology, Key Laboratory of Endocrinology, National Health and Family Planning Commission, Peking Union Medical College Hospital, Chinese Academy of Medical Sciences and Peking Union Medical College, Beijing 100730, China; Department of Endocrinology, Key Laboratory of Endocrinology, National Health and Family Planning Commission, Peking Union Medical College Hospital, Chinese Academy of Medical Sciences and Peking Union Medical College, Beijing 100730, China; Department of Endocrinology, Key Laboratory of Endocrinology, National Health and Family Planning Commission, Peking Union Medical College Hospital, Chinese Academy of Medical Sciences and Peking Union Medical College, Beijing 100730, China; Department of Endocrinology, Key Laboratory of Endocrinology, National Health and Family Planning Commission, Peking Union Medical College Hospital, Chinese Academy of Medical Sciences and Peking Union Medical College, Beijing 100730, China; Department of Endocrinology, Key Laboratory of Endocrinology, National Health and Family Planning Commission, Peking Union Medical College Hospital, Chinese Academy of Medical Sciences and Peking Union Medical College, Beijing 100730, China

**Keywords:** osteogenesis imperfecta, denosumab, bone mineral density, rebound hypercalcemia

## Abstract

**Context:**

Denosumab is a potential therapeutic agent for osteogenesis imperfecta (OI), but its efficacy and safety remain unclear in children with OI.

**Objective:**

We aimed to investigate the effects of denosumab on bone mineral density (BMD), spinal morphometry, and safety in children with OI compared with zoledronic acid.

**Methods:**

In this prospective study, 84 children or adolescents with OI were randomized to receive denosumab subcutaneous injection every 6 months or zoledronic acid intravenous infusion once. Changes of BMD and its Z-score, vertebral shape, serum levels of calcium and bone turnover biomarkers were assessed during the 1-year treatment.

**Results:**

After 12 months of treatment, BMD at the lumbar spine, femoral neck, and total hip significantly increased by 29.3%, 27.8%, and 30.2% in the denosumab group, and by 32.2%, 47.1%, and 41.1% in the zoledronic acid group (all *P* < .001 vs baseline). Vertebral height and projection area significantly increased after denosumab and zoledronic acid treatment. Rebound hypercalcemia was found to be a common and serious side effect of denosumab, of which 14.3% reached hypercalcemic crisis. Rebound hypercalcemia could be alleviated by switching to zoledronic acid treatment.

**Conclusion:**

Treatment with denosumab or zoledronic acid is beneficial in increasing BMD and improving the spinal morphometry of children with OI. However, denosumab should be used with caution in pediatric patients with OI because of its common and dangerous side effect of rebound hypercalcemia. The appropriate dosage and dosing interval of denosumab need to be further explored in children with OI.

Osteogenesis imperfecta (OI) is a rare genetic skeletal disease leading to fractures, bone deformities, scoliosis, and reduced bone mass, with an incidence of 1 in 15 000 to 20 000 in live neonates (1, 2). Patients may also exhibit extraosseous manifestations, including dentinogenesis imperfecta, hearing loss, joint hypermobility, blue sclerae, and cardio/respiratory defects (3). OI is caused by monoallelic mutations in encoding genes of type I collagen, which are autosomal dominant and affect approximately 90% of patients with OI, and the remaining patients are autosomal recessive or X-link inherited due to mutations in genes encoding proteins involved in type I collagen metabolism, bone mineralization, or osteoblastogenesis (1, 3). OI is clinically categorized using the Sillence scale, of which the phenotypes are mild, moderate, and severe for OI types I, IV, and III, and perinatal lethal for OI type II (3, 4). Type V OI is characterized by interosseous membrane ossification and/or hyperplastic callus (3, 4).

The current management of OI mainly focuses on alleviating symptoms, including increasing bone mineral density (BMD), reducing fracture risk, and improving quality of life. Bisphosphonates (BPs) are the most commonly used therapeutic drugs to treat pediatric OI; they can inhibit bone resorption and reduce bone loss. A series of studies has demonstrated that oral (alendronate, risedronate) and intravenous BPs (pamidronate, zoledronic acid [ZOL], neridronate) have beneficial effects on the bones of pediatric patients with OI, such as increasing BMD and reducing the risk of fractures (5–10). Intravenous BPs are the preferred choice for pediatric patients with OI because they are more effective in reducing fracture risk, while oral BPs are mainly used in patients with mild OI (5, 6). Intravenous BPs can also facilitate reshaping of compressed vertebral bodies of children with OI (5, 6, 11, 12). However, BPs cannot change the material properties of bone, and their efficacy in reducing the incidence of fractures has been inconsistent, particularly in patients with severe OI (13). Moreover, gastrointestinal adverse effects of oral BPs, significant acute-phase reactions of intravenous BPs, and poor efficacy on special types of OI with completely different pathophysiology may limit their use in pediatric patients with OI (14–16). Therefore, there is an urgent need to explore new safe and effective therapeutic drugs for patients with OI.

Recently, denosumab, a monoclonal antibody targeting receptor activator of nuclear factor-κB ligand (RANKL), has been approved for the treatment of primary osteoporosis (17). By binding to RANKL, a key mediator of differentiation, function, and survival of osteoclasts, denosumab works through a different pathway from BPs to increase BMD and reduce the fracture incidence in patients with osteoporosis (18, 19). Small-sample studies evaluated the effect of denosumab on the bones of patients with OI, and reported a significant increase in BMD, even in patients with OI type VI who were known to have poor responses to BPs treatment (20–24). Additionally, denosumab treatment is more convenient to use by subcutaneous injection, and there is no long-term bone retention, reducing the possibility of long-term adverse events (25). However, randomized controlled trials comparing the efficacy and safety of denosumab with BPs have not been reported in patients with OI.

Therefore, we prospectively investigate the benefits and risks of denosumab in a relatively large sample of pediatric patients with OI through comparison with ZOL.

## Materials and Methods

### Study Population

Children and adolescents younger than 18 years old were eligible if they fulfilled 1 of the following inclusion criteria: had a history of at least 1 fracture under minor trauma in childhood, and with an age- and gender-adjusted BMD Z-score less than −2.0 at the lumbar spine (LS) or proximal femur; presence of blue sclerae or dentinogenesis imperfecta and with a family history of OI (26); had a clear pathogenic gene mutation of OI identified by targeted next generation sequencing and confirmed by Sanger sequencing (27). Fractures caused by falls below standing heights or fractures in daily activities are defined as fractures under minor trauma.

Exclusion criteria were had unmeasurable BMD at the LS, femoral neck (FN), or total hip; with hypocalcemia; with reduced renal function (estimated glomerular filtration rate (Schwartz formula)<30 mL/min/1.73m^2^); current treatment with other drugs that inhibit bone resorption.

### Study Design

The study was designed as a 1-year, open-label, randomized controlled study. After baseline examinations and screening by endocrinology department of Peking Union Medical College Hospital (PUMCH), children or adolescents with OI were randomized to receive denosumab (Prolia; Amgen Manufacturing Limited, USA) 30 mg or 60 mg subcutaneous injection every 6 months (21) or ZOL (Aclasta; Novartis Pharma SchweizAG, Switzerland) 2.5 mg or 5 mg intravenous infusion once (26). Children over 5 years old were given denosumab at a dose of 60 mg every 6 months, and those under 5 years old received a half dose of denosumab (30 mg every 6 months) (21). ZOL was administered at a dose of 5 mg (in children with a body weight >25 kg) or 2.5 mg (in children with a body weight ≤25 kg) once a year (26). Computer-generated random permuted blocks were used, stratified by age and gender. All patients were supplemented daily with calcium (600 mg) and 125 IU vitamin D (Caltrate D; Wyeth Pharmaceuticals, Madison, WI, USA) for 1 year; the daily dosage would be halved (300 mg of calcium and 62.5 IU vitamin D) in patients less than 5 years old. Calcitriol (0.25 μg, Rocaltrol; R.P. Scherer GmbH & Co. KG, Germany) was administered on alternate days for all patients for 1 year. If hypercalcemia occurred, calcium, vitamin D, and calcitriol would be stopped immediately.

The primary endpoints were the changes in BMD at the LS, FN and total hip during the treatment. The secondary endpoints were the changes in serum concentrations of calcium and bone turnover biomarkers, the incidence of new fractures, and the changes in spinal morphometry. The study was conducted in accordance with the principles of the Declaration of Helsinki and was approved by the Scientific Ethics Committee of PUMCH (Approval Number: JS-2081). The parents or legal guardians of all patients with OI provided written informed consent before they participated in this study.

### Measurement of Bone Mineral Density

Areal bone mineral density (aBMD) at the LS, FN, and total hip were measured by dual-energy X-ray absorptiometry (DXA; Lunar Prodigy Advance, GE Healthcare, USA) at baseline, 6 and 12 months of treatment. To ensure the accuracy of the DXA measurements, phantom testing was performed daily for calibration and quality checks. The coefficients of variation for DXA measurements were 1.1% and 1.7% at LS and FN, respectively. Based on the aBMD reference data of normal children in China (28), the Z-scores of aBMD at LS and FN were calculated.

### Assessment of Spinal Morphometry and Vertebral Compression Fracture

Lateral thoracolumbar films were taken at baseline, and 6 and 12 months of treatment. According to the thoracolumbar films, the morphometry of the fourth thoracic vertebra to the fourth lumbar vertebra was quantitatively measured at baseline and 12 months of treatment by a radiologist (8). The 4 corners and 4 midpoints of the endplate were marked on each vertebra, and the anterior, posterior, and midheight (ah, ph, and mh) and upper, lower, and midlength (UL, LL, and ML) were determined using the DICOM system with an accuracy of 0.1 mm. The concavity index was calculated as mh/ph and the height to length ratios were calculated as ah/LL, mh/LL, and ph/LL to assess the degree of vertebral compression (8). The projection area for each vertebra was calculated. Moreover, vertebral compression fracture was assessed according to the Genant semiquantitative grading scale (29).

The body height of the patients was measured using a Harpenden stadiometer (Seritex Inc., East Rutherford, NJ, USA) at baseline, 6 and 12 months of treatment. For patients who could not stand, supine body length was measured. The body height Z-scores were calculated based on the reference data of Chinese children (30). The Z-scores of body mass index were calculated based on growth curves for Chinese children (31).

### Assessment of Bone Metabolic Indexes

Blood samples of patients with OI were collected between 8:00 Am and 10:00 Am after overnight fasting at baseline, 1, 6, and 12 months of treatment. Serum concentrations of procollagen type I N-terminal propeptide (a bone formation marker), β-isomerized carboxy-telopeptide of type I collagen (β-CTX, a bone resorption marker), 25-hydroxyvitamin D, and parathyroid hormone were measured using an automated electrochemiluminescence system (E170, Roche Diagnostics, Switzerland). Serum levels of calcium (Ca), phosphate (P), alanine aminotransferase, creatinine, and alkaline phosphatase (a bone formation marker) were detected by automated analyzers (ADVIA1800, Siemens, Germany). All biochemical indexes were measured uniformly at the central laboratory of PUMCH.

Rebound hypercalcemia was considered when serum calcium levels exceeded the normal upper limit during denosumab treatment and no other reasons for hypercalcemia were found. Hypercalcemia is classified as mild or moderate if serum calcium level is between 2.63 and 3.00 mmol/L (10.5 and 12.0 mg/dL), or between 3.00 and 3.50 mmol/L (12.0 and 14.0 mg/dL), respectively (32). Hypercalcemic crisis is defined when serum calcium level is higher than 3.50 mmol/L (14.0 mg/dL), which will be life-threatening and requires urgent treatment (33).

### Assessment of the Safety of Treatment

Laboratory examinations were performed, including repeated calcium level determinations. In addition to standard reporting of all adverse events, clinical data were collected during each follow-up. Serious adverse events included severe infection, osteonecrosis of the jaw, metaphyseal osteosclerosis, new fracture, severe biochemical abnormalities, hospitalization, and even death.

### Statistical Analyses

All analyses were conducted using full intention to treat set and per-protocol set. Continuous data of normal distribution were presented as mean ± SD and were compared by Student's t-test between the denosumab and ZOL groups. Continuous data of abnormal distribution were shown as median (interquartile range, IQR) and were compared using Mann–Whitney U test between the denosumab and ZOL groups. We completed paired samples t-test for normally distributed variables and Wilcoxon paired test for abnormally distributed variables to assess the changes from baseline to 6 and 12 months of treatment. Categorical data were expressed as numbers and percentages (%) and were compared using Pearson's chi-square test.

*P* < .05 was considered to be statistically significant. Statistical analyses were performed using SPSS statistics software for Windows (version 25.0; IBM Corp., Armonk, NY, USA). Graphs were produced using GraphPad Prism software version 8.4.2 (GraphPad Software, La Jolla, CA, USA).

## Results

### Baseline Characteristics of the Patients

**Figure 1.**
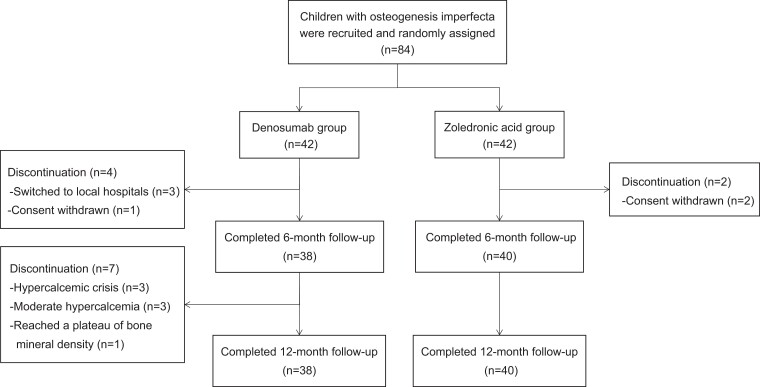
Flow chart of this study.

The study cohort included 84 pediatric patients with OI, with 42 patients in each group of denosumab or ZOL (Fig. 1). The mean ages were 7.4 years (range 0.2-15.5) and 7.6 years (range 2.0-16.7) in the denosumab and the ZOL groups, respectively. The mean weights of patients with OI were 24.7 kg (range 4.0-61.0) and 28.1 kg (range 11.0-80.0) in the denosumab and the ZOL groups, respectively. Serum levels of 25-hydroxyvitamin D (median [IQR]) were 30.7 (22.4, 44.3) ng/mL in the denosumab group and 24.0 (18.4, 33.5) ng/mL in the ZOL group at baseline, and no significant difference was found between the 2 groups. A synopsis of baseline characteristics was shown in Table 1, and no significant differences were found between denosumab and ZOL groups. The causative mutation profiles of OI were as follows: *COL1A1* (n = 16, 38.1%), *COL1A2* (n = 10, 23.8%), *IFITM5* (n = 3, 7.1%), *WNT1* (n = 3, 7.1%), *P3H1* (n = 1, 2.4%), *PLOD2* (n = 1, 2.4%), and no detected mutations (n = 8, 19.0%) in the denosumab group; *COL1A1* (n = 22, 52.4%), *COL1A2* (n = 9, 21.4%), *IFITM5* (n = 2, 4.8%), mutations in *PLS3*, *CRTAP*, *FKBP10*, *PLOD2* and *WNT1* (1 for each gene mutation, 2.4%), and no detected mutations (n = 4, 9.5%) in the ZOL group.

**Table 1.** Baseline characteristics of patients with OI

Characteristic	Denosumab group (n = 42)	Zoledronic acid group (n = 42)	Reference
Male:Female	27:15	27:15	
Clinical type of OI (I:III:IV:V)	14:14:11:3	20:8:12:2	
Age (years), mean (SD)	7.35 (4.16)	7.57 (4.11)	
Height (cm)^*a*^, mean (SD)	121.5 (26.1)	119.4 (24.2)	
Height Z-score, mean (SD)	−1.367 (2.033)	−0.968 (2.110)	
Weight (kg)*^a^*, mean (SD)	24.7 (15.0)	28.1 (17.3)	
Weight Z-score, mean (SD)	−0.552 (1.518)	−0.033 (1.376)	
BMI (kg/m^2^)*^b^*, mean (SD)	17.3 (4.2)	17.4 (4.1)	
BMI Z-score, mean (SD)	0.157 (1.936)	−0.237 (1.223)	
Previous fractures times, median (IQR)	4 (3, 6)	4 (2, 5)	
Patients with previous vertebral fractures (%)	9 (21.4)	14 (33.3)	
Patients with previous use of bisphosphonates (%)	6 (14.3)	8 (19.0)	
ALT (U/L), median (IQR)	13 (11, 19)	13 (11, 16)	5 ∼ 90
Cr (E) (μmol/L), median (IQR)	30 (26, 37)	34 (27, 39)	59 ∼ 104
Ca (mmol/L), mean (SD)	2.49 (0.09)	2.45 (0.10)	2.13 ∼ 2.70
P (mmol/L), mean (SD)	1.64 (0.19)	1.60 (0.24)	1.29 ∼ 1.94
ALP (U/L), mean (SD)	311 (104)	304 (106)	58 ∼ 400
β-CTX (ng/mL), mean (SD)	1.06 (0.31)	0.97 (0.33)	0.21 ∼ 0.44
25OHD (ng/mL), median (IQR)	30.7 (22.4, 44.3)	24.0 (18.4, 33.5)	20.0 ∼ 50.0
PTH (pg/mL), median (IQR)	26.1 (20.3, 31.2)	23.3 (16.8, 31.1)	15.0 ∼ 65.0
LS aBMD (g/cm^2^)*^c^*, mean (SD)	0.500 (0.185)	0.541(0.173)	
LS aBMD Z-score, mean (SD)	−1.501 (1.844)	−0.953 (2.041)	
FN aBMD (g/cm^2^)*^c^*, mean (SD)	0.461 (0.151)	0.487 (0.177)	
FN aBMD Z-score, mean (SD)	−3.112 (1.958)	−2.587 (2.490)	
TROCH aBMD (g/cm^2^), mean (SD)	0.377 (0.137)	0.409 (0.229)	
TH aBMD (g/cm^2^), mean (SD)	0.479 (0.148)	0.513 (0.166)	

No significant differences in baseline characteristics were found between denosumab and zoledronic acid groups. Values are given as number (proportion), mean (SD) or median (IQR).

Abbreviations: β-CTX, β-isomerized carboxy-telopeptide of type I collagen; aBMD, areal bone mineral density; ALP, alkaline phosphatase; ALT, alanine aminotransferase; BMI, body mass index; Ca, calcium; Cr, creatinine; FN, femoral neck; IQR, interquartile range.; LS, lumbar spine; OI, osteogenesis imperfecta; ; P, phosphate; PTH, parathyroid hormone; SD, standard deviation; TH, total hip; TROCH, trochanter; 25OHD, 25-hydroxyvitamin D.

^*a*^The reference ranges for height and weight of different ages were based on standardized growth charts for Chinese children and adolescents (30).

^*b*^The reference ranges for BMI of different ages were based on growth curves for Chinese children and adolescents (31).

^*c*^The reference ranges for aBMD at the lumbar spine and femoral neck of different ages were based on a large-sample cross-sectional study in China (28).

A total of 71 patients completed the study. Eleven patients in the denosumab group terminated the treatment early because of hypercalcemia (n = 5), reluctance to continue treatment (n = 1), willingness to refer to local hospitals (n = 3), achievement of normal aBMD and switching to other treatments (n = 1), and loss to follow-up due to COVID-19 (n = 1). Two patients in the ZOL group were lost to follow-up because of COVID-19. In total, 4 patients in the denosumab group and 2 patients in the ZOL group were lost to follow-up (Fig. 1).

### Primary Endpoints

#### Changes of areal bone mineral density

**Figure 2.**
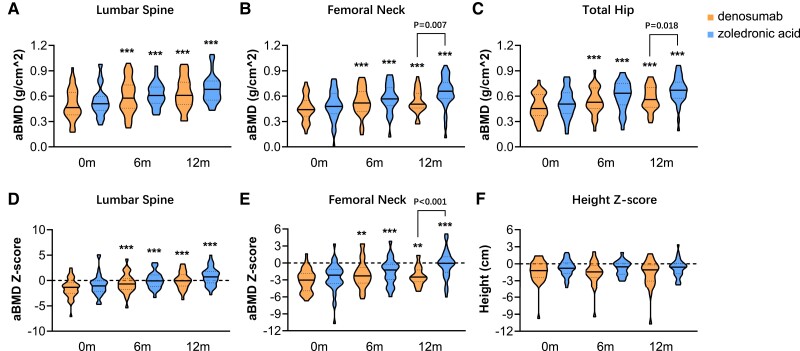
Changes in the areal bone mineral density (aBMD), Z-score, and height Z-score after denosumab or zoledronic acid treatment. A, Changes in aBMD at the lumbar spine. B, Changes in aBMD at the femoral neck. C, Changes in aBMD at the total hip. D, Changes in aBMD Z-score at the lumbar spine. E, Changes in aBMD Z-score at the femoral neck. F, Changes in height Z-score. A-F, Are presented as violin plots with median (solid line) and interquartile range (IQR, dotted line). **P < .01, ***P < .001 vs baseline. m, month.

All patients were included in the intention to treat analysis. After 12 months of treatment, aBMD at the LS, FN, and total hip significantly increased by 29.3%, 27.8%, and 30.2% in the denosumab group and by 32.2%, 47.1%, and 41.1% in the ZOL group (all *P* < .001 vs baseline) (Fig. 2A-2C). The increases in aBMD at the FN and total hip in the denosumab group were lower than those in the ZOL group (all *P* < .05) (Fig. 2B and 2C), with less increase in age-adjusted aBMD Z-score at the FN in the denosumab group than that in the ZOL group (*P* < .001) (Fig. 2E).

### Secondary Endpoints

#### Changes of serum calcium and bone turnover biomarkers

**Figure 3.**
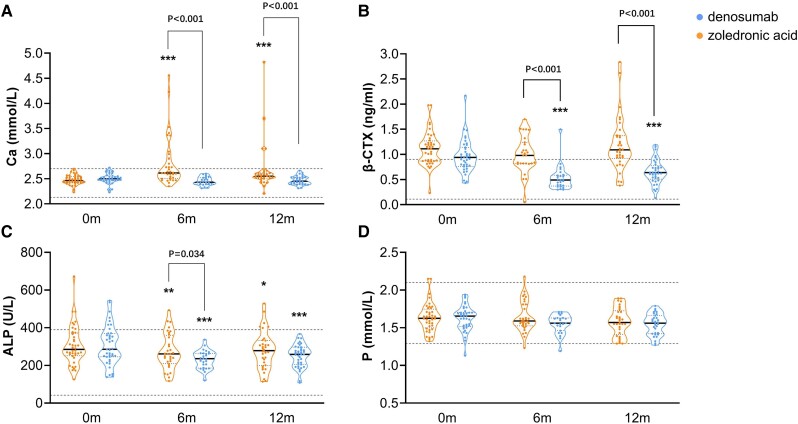
Changes in biochemical markers after denosumab or zoledronic acid treatment. A, Changes in serum concentrations of Ca. B, Changes in serum concentrations of β-CTX. C, Changes in serum concentrations of ALP. D, Changes in serum concentrations of phosphate. A-D, Are presented as violin plots with median (solid line) and interquartile range (IQR, dotted line). Two gray broken lines in each graph represent the normal upper and lower limit of biochemical markers, respectively. The points exceeding the normal upper limit in Fig. 3A represent serum concentrations of calcium in patients with hypercalcemia. Ca, calcium; β-CTX, β-isomerized carboxy-telopeptide of type I collagen; ALP, alkaline phosphatase. *P < .05, **P < .01, ***P < .001 vs baseline. m, month.

Serum Ca concentrations significantly increased after 6 and 12 months of denosumab treatment (all *P* < .001 vs baseline) (Fig. 3A). No significant differences in serum Ca levels were found during 1 year of ZOL treatment (Fig. 3A). Rebound hypercalcemia was found in 13 patients from the denosumab group (31.0%), of whom 6 patients (46.2%) had a hypercalcemic crisis: 5 patients and 2 patients had moderate and mild hypercalcemia, respectively. Clinical signs of hypercalcemia included poor appetite, nausea, vomiting, thirst, and polyuria. The mean time to onset of rebound hypercalcemia was 4.7 months since the last injection of denosumab, and it could appear as early as 3.1 months after denosumab treatment (Fig. S1) (34)). In this cohort, rebound hypercalcemia was more likely to occur in children younger than 5 years of age with OI (80.0%, *P* < .001 vs children older than 5 years), and serum β-CTX levels were significantly higher in patients with hypercalcemia than in those without hypercalcemia (1.65 ± 0.77 vs 0.97 ± 0.39, *P* < .01) (Table 2).

**Table 2.** Comparison of patients with hypercalcemia and without hypercalcemia after denosumab treatment

Variable	With hypercalcemia (n = 13)	Without hypercalcemia (n = 29)
Age (years), mean (SD)	3.0 (2.3)	9.5 (3.2)***
Proportion in children <5 years, n (%)	12 (80.0)	3 (20.0)***
Proportion in children >5 years, n (%)	1 (3.7)	28 (96.3)***
Male:Female	7:6	20:9
Clinical type of OI (I:III:IV:V)	5:5:2:1	9:9:9:2
Ca (mmol/L), median (IQR)	3.39 (3.03, 3.86)	2.54 (2.47, 2.61)***
Hypercalcemia crisis, n (%)	6 (46.2)	/
P (mmol/L), mean (SD)	1.67 (0.33)	1.63 (0.18)
ALP (U/L), mean (SD)	289 (109)	240 (81)
β-CTX (ng/mL), mean (SD)	1.65 (0.77)	0.97 (0.39)**
25OHD (ng/mL), median (IQR)	48.9 (32.4, 64.2)	23.3 (17.3, 29.0)**
PTH (pg/mL), mean (SD)	10.7 (5.2)	24.9 (11.3)***
ALT (U/L), median (IQR)	13 (12, 17)	15 (11, 26)
Cr(E) (μmol/L), median (IQR)	36 (24, 42)	33 (29, 43)

Values are given as number (proportion), mean (SD) or median (IQR).

Abbreviations: ALP, alkaline phosphatase; ALT, alanine aminotransferase; Ca, calcium; Cr, creatinine; IQR, interquartile range; OI, osteogenesis imperfecta; P, phosphate; PTH, parathyroid hormone; SD, standard deviation; 25OHD, 25-hydroxyvitamin D; β-CTX, β-isomerized carboxy-telopeptide of type I collagen.

*P < .05, **P < .01, ***P < .001 vs OI with hypercalcemia.

Once hypercalcemia occurred in the denosumab group, calcium, vitamin D, and calcitriol were stopped and 2.5 mg or 5 mg of ZOL was intravenously infused immediately. Two patients with hypercalcemic crisis were admitted to local hospitals, including a 1.9-year-old girl with a serum Ca level of 4.56 mmol/L (18.24 mg/dL) after 5.6 months since the second injection of denosumab and a 0.7-year-old boy with serum Ca level of 4.83 mmol/L (19.32 mg/dL) after 5 months since the second injection of denosumab. They were immediately given an intravenous infusion of 2.5 mg of ZOL and a large amount of fluid, and discontinuation of calcium carbonate, vitamin D, and calcitriol. The remaining 4 patients with hypercalcemic crisis received the same treatment and were closely monitored in the emergency department. After the above administration, the serum Ca levels returned to normal range within 1 week, and hypercalcemia did not appear again at observation during the following year. The patients with mild or moderate hypercalcemia received the same treatment as above and were monitored in the endocrinology clinic. Their serum Ca levels returned to normal range within 1 week.

Serum β-CTX levels significantly decreased after 12 months of ZOL treatment (*P* < .001 vs baseline), but there were no significant differences in serum β-CTX levels between baseline and at 6 and 12 months of denosumab treatment (Fig. 3B). Serum alkaline phosphatase levels were significantly decreased during denosumab (*P* < .05) and ZOL treatment (*P* < .001) (Fig. 3C). Serum parathyroid hormone levels significantly decreased after 12 months of denosumab treatment (*P* < .001 vs baseline), but there were no obvious changes during ZOL treatment.

#### New fracture incidence

During treatment, 9 patients (21.4%) in the denosumab group and 7 patients (16.7%) in the ZOL group developed new nonvertebral fractures, of which femoral fractures accounted for 50.0% (n = 8), followed by tibial fractures (n = 3), humeral fractures (n = 3), and calcaneus and clavicle fractures (1 case each). Only 1 patient (2.4%) in the ZOL group had a new vertebral fracture. No delayed healing of fractures was observed in the 2 groups.

#### Changes of spinal morphometry

**Figure 4.**
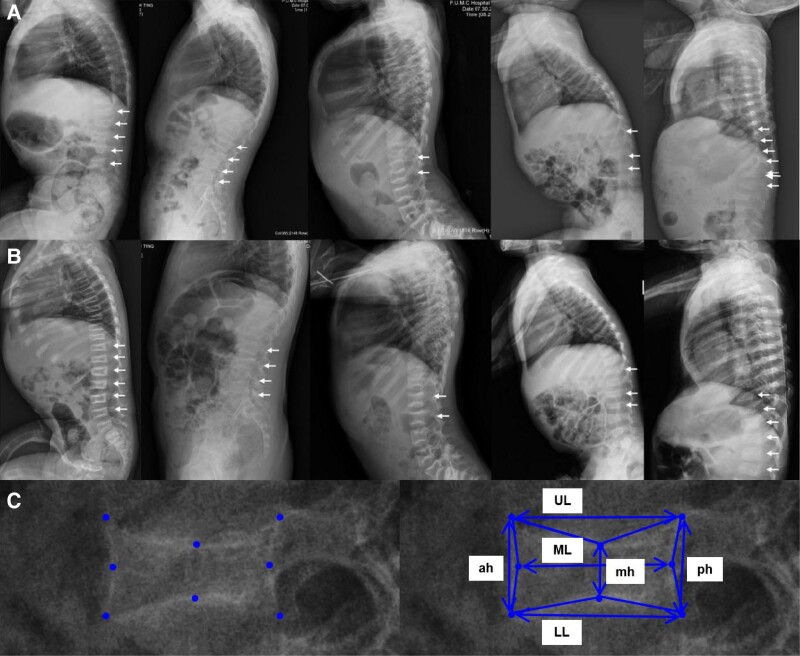
Reshape of fractured vertebrae after denosumab treatment. A, Thoracolumbar lateral films at baseline. B, Thoracolumbar lateral films after 12-month denosumab treatment. C, Quantitative measurement of vertebral morphometry on lateral films. White arrows showed the reshape of fractured vertebrae after denosumab treatment. Ah, anterior height of vertebra; mh, middle height of vertebra; ph, posterior height of vertebra; UL, upper length; ML, middle length; LL, lower length. Lateral projection area = Area 1 + Area 2 + Area 3 + Area 4. Area 1 = [(ah/2) × (UL/2)] − [((ah/2) × ((UL − ML)/2))/2] − [((UL/2) × ((ah − mh)/2))/2]. Area 2 = [(ph/2) × (UL/2)] − [((ph/2) × ((UL − ML)/2))/2] − [((UL/2) × ((ph − mh)/2))/2]. Area 3 = [(LL/2) × (ah/2)] − [((ah/2) × ((LL − ML)/2))/2] − [((LL/2) × ((ah − mh)/2))/2]. Area 4 = [(LL/2) × (ph/2)] − [((ph/2) × ((LL − ML)/2))/2] − [((LL/2) × ((ph − mh)/2))/2].

The vertebral shape was quantitatively assessed in 38 and 40 patients in the denosumab and ZOL groups at baseline and after 12 months of treatment. At baseline, no obvious differences were found in spinal morphometry between the 2 groups. After 12 months of treatment, the average projection area, mh/ph, and mh/LL significantly increased in the denosumab group (Fig. 4), and the average projection area significantly increased in the ZOL group (Table 3). The average body height of children with OI increased by 5.6 and 5.7 cm after 12 months of denosumab and ZOL treatment (all *P* < .001 vs baseline), which were similar in the 2 groups. Height Z-scores did not improve in the 2 groups (Fig. 2F), indicating no catch-up growth during the 1-year treatment of denosumab or ZOL.

**Table 3.** Changes in the vertebral morphometric parameters after denosumab or zoledronic acid treatment

Vertebral morphometry	Denosumab group (n = 38)		Zoledronic acid group (n = 40)	
	Baseline	12 months	Baseline	12 months
Average PA (mm^2^), mean (SD)	325.15 (155.68)	380.75 (172.39)***	313.98 (136.35)	356.86 (164.26)**
Average mh/ph, mean (SD)	0.84 (0.18)	0.87 (0.16)*	0.94 (0.13)	0.94 (0.15)
Average ah/LL, mean (SD)	0.56 (0.10)	0.59 (0.12)	0.56 (0.08)	0.56 (0.06)
Average mh/LL, mean (SD)	0.56 (0.13)	0.61 (0.14)***	0.61 (0.12)	0.62 (0.10)
Average ph/LL, mean (SD)	0.65 (0.11)	0.67 (0.10)	0.65 (0.07)	0.66 (0.06)

Values are given as mean (SD).

Abbreviations: ah, anterior height of vertebra; LL, lower length; mh, middle height of vertebra; PA, projection area; ph, posterior height of vertebra; SD, standard deviation.

*P < .05, **P < .01, ***P < .001 vs baseline.

#### Safety

Sixteen (38.1%) and 34 patients (81.0%) in the denosumab and ZOL groups had adverse events (Table 4). Hypercalcemia was more common in the denosumab group than in the ZOL group (31.0% vs 0%, *P* < .001), and acute-phase reactions, such as fever, were more frequent in the ZOL group than in the denosumab group (71.4% vs 0%, *P* < .001). Two patients in the denosumab group were hospitalized because of hypercalcemic crisis. Nine patients in the denosumab group and 8 patients in the ZOL group developed new fractures. Osteosclerosis was found in 1 patient in the denosumab group characterized by a club-shaped and radiodense appearance at the proximal femoral, distal femoral, and proximal tibial metaphyses (Fig. S2 (34, 35)). Other adverse events were similar between the 2 treatment groups (Table 4). No hepatic or renal dysfunction, hypocalcemia, severe infection, osteonecrosis of the jaw was observed. No impairment of consciousness or deaths occurred.

**Table 4.** Adverse events in the denosumab and zoledronic acid groups

Adverse events	Denosumab group (n = 42)	Zoledronic acid group (n = 42)
New fractures, n (%)	9 (21.4)	8 (19.0)
Fever, n (%)	4 (9.5)***	30 (71.4)
Musculoskeletal pain, n (%)	9 (21.4)	4 (9.5)
Infection, n (%)	0 (0)	0 (0)
Hypocalcemia, n (%)	1 (2.4)	0 (0)
Symptoms of hypercalcemia, n (%)	13 (31.0)***	0 (0)
Thirst	6 (14.3)*	0 (0)
Polyuria	5 (11.9)	0 (0)
Poor appetite	10 (23.8)**	0 (0)
Nausea and vomiting	6 (14.3)*	0 (0)
Constipation	1 (2.4)	0 (0)
Hepatic dysfunction, n (%)	0 (0)	0 (0)
Renal dysfunction, n (%)	0 (0)	0 (0)
Osteonecrosis of the jaw, n (%)	0 (0)	0 (0)
Metaphyseal osteosclerosis, n (%)	1 (2.4)	0 (0)
Serious adverse events, n (%)	10 (23.8)	8 (19.0)
Hypercalcemia crisis, n (%)	6 (14.3)*	0 (0)
Death, n (%)	0 (0)	0 (0)

Values are given as number (proportion).

*P < .05, **P < .01, ***P < .001 vs zoledronic acid.

## Discussion

Reports on denosumab application in patients with OI are very rare. We investigated the safety and efficacy of denosumab in a large cohort of children with OI through comparison with ZOL. We found that denosumab not only significantly increased aBMD and its Z-score of children with OI, but also significantly improved the patients’ spinal morphometry. However, rebound hypercalcemia was a quite common and serious adverse event of denosumab in children with OI (31%), of whom 46.2% even developed hypercalcemic crisis. We found that young age and high bone resorption level were risk factors for rebound hypercalcemia in children with OI during denosumab treatment.

It is important to treat bone fragility to reduce the risk of bone fractures, which is beneficial for improving the quality of life of patients with OI. Intravenous BPs are the most broadly used agents for patients with OI because they can inhibit bone resorption, increase BMD, and improve the spinal shape of pediatric patients with OI. However, there were inconsistent outcomes on the effect of BPs in reducing the incidence of fractures (10, 13, 15, 36–38). A recent study indicated that patients with OI with pathogenic mutations leading to collagen structural defects or with nonautosomal dominant inheritance may have relatively poor responses to ZOL treatment (26). Here we investigate the efficacy of ZOL in a dose indicated to be generally safe for children with OI in previous studies (8, 26). The results of these studies revealed the current dose was effective in increasing aBMD and its Z-score, as well as improving the vertebral shape, while further long-term research is still needed to examine its efficacy in reducing fracture rate. Therefore, new antiresorptive drugs, such as denosumab, and anabolic agents need to be investigated in patients with OI.

Denosumab can inhibit osteoclast activity, reduce bone degradation, and increase BMD, which has been approved for the treatment of postmenopausal osteoporosis, male osteoporosis, and glucocorticoid-induced osteoporosis (18, 39, 40). Recent series of studies reported that subcutaneous injections of denosumab showed promising results in small samples of children with OI. A retrospective study indicated that LS and hip BMD of 8 pediatric or adult patients with OI significantly increased after 4 to 54 months of denosumab (15, 30, or 60 mg every 6 months) treatment, without severe side effects occurring (21). A 2-year study showed that treatment with denosumab (1 mg/kg body weight every 12 weeks) increased BMD, promoted vertebral body reshaping, improved movement function, and reduced fracture rate in patients with type VI OI (23). A prospective study suggested that 48 weeks of denosumab treatment (1 mg/kg body weight every 12 weeks) significantly increased LS BMD of 10 pediatric patients with OI (20). As the sample size of these studies was quite small, and the dosage of denosumab used in partial studies was not uniform, it was difficult to clarify the effectiveness and safety of denosumab in patients with OI.

In this study, we prospectively evaluated the effects of denosumab and ZOL on bone metabolism and calcium homeostasis in a relatively large OI pediatric cohort. The participants received denosumab treatment according to the regimen from a Japanese study (21). Our results indicated that denosumab was beneficial in increasing aBMD and improving the spinal morphometry of children with OI, which were similar to the outcomes of previous small sample studies (20, 21, 23). Moreover, to minimize the impact of growth on aBMD as much as possible, we also evaluated the changes in aBMD Z-score and observed significant increases in aBMD Z-scores at the LS and FN during treatment. In this study, we found significant increases in average vertebral height and projection area of patients with OI after 12-month denosumab treatment, indicating that vertebral reshaping occurred, and the results were consistent with the effect of ZOL (8). After treatment with denosumab and ZOL, a patient's height significantly increased, but no catch-up growth was observed. Notably, the current dose of denosumab significantly improved bone phenotypes of OI, but was associated with serious rebound hypercalcemia. In this study, the side effect of rebound hypercalcemia was quite common in children with OI during denosumab treatment, and some patients even experienced a life-threatening hypercalcemic crisis. We found that young age and high bone resorption level were risk factors for rebound hypercalcemia. Additionally, metaphyseal osteosclerosis was observed in 1 patient receiving denosumab treatment, which was similar to that in children with OI who received ZOL treatment (35). This phenomenon may be related to increased hypermineralized bone in the metaphyseal region (41). It was reported that metaphyseal osteosclerosis can be resolved after stopping BPs therapy (42). The child with metaphyseal osteosclerosis in our study requires further follow-up.

Information on the safety of denosumab in growing children is very scarce. In this study, rebound hypercalcemia was observed in 31.0% of patients with OI at an average of 4.7 months since the last denosumab injection. Rebound hypercalcemia consequent to denosumab administration was reported for the first time in a juvenile patient with fibrous dysplasia. This patient experienced severe hypercalcemia and a dramatic rebound of bone turnover was found after 3 months of denosumab treatment (43). Previous research has provided a glimpse of the safety concerns regarding rebound hypercalcemia. In a case series of 8 patients with OI, mild hypercalcemia was recorded 6 months after a single dose of 15 mg of denosumab in a 3-year-old girl (21). In another study, 4 children with OI type VI were treated with the regimen of 1 mg/kg denosumab every 3 months, and 2 patients aged 3.9 and 4.6 years developed hypercalcemia (ionized calcium of 1.54 and 1.62 mmol/L) between 7 and 12 weeks after the preceding denosumab injection (24). In our study, rebound hypercalcemia was quite common, and young age and high bone resorption level were its risk factors. The dosage of denosumab and its administration interval in our study was the same as a Japanese study (21), but it was different from the other studies (20, 23), which could also be associated with more frequent rebound hypercalcemia in our patients. Therefore, denosumab treatment should be considered as an alternative to intravenous BP therapy in pediatric patients with OI, but it needs to be used with caution. It is necessary to explore the appropriate dosage and dosing interval of denosumab based on age and body weight of pediatric patients with OI.

As rebound hypercalcemia, especially hypercalcemic crisis, is a serious adverse event of denosumab, it is important to monitor serum calcium levels closely and dynamically in pediatric patients with OI during denosumab treatment. To improve bone condition and prevent possible hypocalcemia, children with OI were supplemented with calcitriol and calcium, which did not cause hypercalcemia in children with OI treated with ZOL (26, 44). However, supplementation with calcitriol and calcium could involve in the high incidence of hypercalcemia during denosumab treatment. Therefore, active vitamin D analogs and calcium should be used cautiously in children with OI during denosumab treatment, especially in young children with OI. In this study, infusion of ZOL, adequate hydration, and discontinuation of calcium and calcitriol were effective in relieving denosumab-induced rebound hypercalcemia. After the above treatment, rebound hypercalcemia did not recur during follow-up for 1 year. As ZOL could reduce the activated bone resorption caused by denosumab, it could effectively control rebound hypercalcemia for a long time (45, 46). In addition to identifying high bone turnover as a risk factor for rebound hypercalcemia, we observed no reduction in bone resorption levels after denosumab treatment. This finding differed from the effects observed with ZOL treatment but was consistent with previous research (24). The discrepancy may be attributed to the short action time of denosumab, which can result in a rebound of bone resorption and bone loss at the end of the interval before their next dose of denosumab (47). Therefore, denosumab must be used with caution in young children, and further studies are needed to investigate the right timing and dose regimens of ZOL for the treatment and prevention of rebound hypercalcemia.

In this large cohort of children with OI, we prospectively found that denosumab treatment was beneficial in increasing aBMD and improving spinal morphometry, but there was a high risk of rebound hypercalcemia. Our study provided valuable clinical experience for the application of denosumab in pediatric patients with OI. However, there were a series of limitations in this study. At the beginning of this study, the dosage of denosumab for the treatment of children with OI was still in the exploratory stage. Thus, we selected the therapeutic dose based on previous studies (8, 21). This was a limitation as we did not choose weight-based dosing of denosumab, making it difficult to compare the effects of weight-adjusted doses of denosumab and ZOL. In the future, we will conduct studies using weight-based doses of denosumab and ZOL to observe the safety and efficacy of the treatment in children with OI (12, 24). The treatment period was only 1 year in this study, so the impact of denosumab treatment on fracture incidence was difficult to observe, and the post-trial follow-up period should be taken into account to evaluate the long-term risk and benefit ratio of denosumab in children with OI. Furthermore, we did not intensively and dynamically monitor the changes in serum levels of calcium and bone turnover biomarkers. The incidence of asymptomatic hypercalcemia may be underestimated, and the specific initiation time and change patterns of hypercalcemia during denosumab treatment remain unclear. Dynamic alterations in biomarkers of bone turnover may also fail to be captured during denosumab treatment. Renal ultrasonography was not performed in patients with hypercalcemia. The sample size was not large enough to analyze the relationship between the genotype and the response of patients with OI to denosumab treatment.

In conclusion, treatment with denosumab can significantly increase BMD and improve the spinal morphometry of pediatric patients with OI, which is similar to the efficacy of ZOL. However, rebound hypercalcemia is a quite common and severe side effect of denosumab, which makes denosumab not an option for children with OI as first-line treatment. Young age and high bone resorption level are risk factors for rebound hypercalcemia, and intravenous ZOL can effectively alleviate this side effect. In the future, the appropriate weight-based dosage and dosing interval of denosumab need to be explored in pediatric patients with OI.

## Data Availability

Restrictions apply to the availability of some or all data generated or analyzed during this study to preserve patient confidentiality or because they were used under license. The corresponding author will on request detail the restrictions and any conditions under which access to some data may be provided.

## Abbreviations

β-CTX: β-isomerized carboxy-telopeptide of type I collagen
BMD: bone mineral density
BP: bisphosphonate
DXA: dual-energy X-ray absorptiometry
FN: femoral neck
LS: lumbar spine
OI: osteogenesis imperfecta
ZOL: zoledronic acid
